# Motives and modifying factors for giving or rejecting psychiatric diagnoses in general medicine and psychiatry – a qualitative interview study

**DOI:** 10.1186/s12888-024-05900-2

**Published:** 2024-06-20

**Authors:** Hannah Tebartz van Elst, Claudia Niehoff, Jost Steinhäuser

**Affiliations:** Institute of Family Medicine, University Medical Center Schleswig-Holstein, Ratzeburger Allee 160, 23538 Campus Lübeck, Germany

**Keywords:** Qualitative study, Psychiatric diagnoses, General medicine, Psychiatry, Clinical reasoning

## Abstract

**Background:**

There is a discussion among general practitioners and psychiatrists regarding over-diagnosing versus under-reporting of psychiatric diagnoses. A deeper understanding of this topic is relevant for providing reasonable health care and for planning future studies. A crucial factor to understanding this discussion is the difference in the prevalence of a disease in each sector. One way to attain knowledge about such prevalences is the analysis of routine care data of the sector in question. However, diagnosis-related data might be modified by several additional influencing factors.

**Aims:**

This study aims to explore what kind of motives and modifying factors play a role for or against giving psychiatric diagnoses in psychiatric and general medical settings.

**Methods:**

Twenty-six semi-structured interviews were conducted with German physicians in the fields of general medicine and psychiatry. Interviews were analysed using content analysis.

**Results:**

The analysis revealed three major motivational categories for finding a diagnosis: (1) “objective matters” such as “categorisation for research”; (2) “functional and performance-related factors” such as “requirement for medication”, “billing aspects” that go with certain diagnoses or “access to adequate care” and (3) “Individual factors” such as the “personality of a physician”. Similarly, factors emerged that lead to not making psychiatric diagnoses like “fear of stigmatization among patients” or “detrimental insurance status with psychiatric diagnosis”. Additionally participants mentioned other reasons for “not diagnosing a psychiatric diagnosis“, such as “coding of other clinical pictures”.

**Conclusion:**

The diagnostic process is a complex phenomenon that goes far beyond the identification of medical findings. This insight should be considered when processing and interpreting secondary data for designing health care systems or designing a study.

**Supplementary Information:**

The online version contains supplementary material available at 10.1186/s12888-024-05900-2.

## Background

It is generally assumed that a medical diagnosis identifies a fact or names a condition or a disease, which then forms the basis for further treatment. In practice, however, the process of making a diagnosis is not exclusively based on empirical facts but depends on multiple factors. They may depend on the disease concept of the physician or of the patient, the physician’s overall diagnostic goal, the physician’s age and general diagnostic style and behavior, the consideration of what the impact of the diagnosis on the patient might be and aspects of the health care system [1–6].

Other relevant aspects might also influence a diagnosis, such as concerns about causing fear in patients or the fear of stigmatizing consequences [7, 8]. Furthermore, the problem of “diagnostic uncertainty”, which may lead to a diagnosis not being made is also known [9].

Psychiatric diseases are of particular relevance, since their definition and detection are complex and more affected by the personal beliefs and attitudes of physicians and the background of patients [4, 10–13].

The majority of people with mental health problems and potentially diagnosable mental disorders see and are treated by general practitioners (GPs) [14]. Studies show that in Germany, depending on the psychiatric diagnosis, 55–97% of patients with mental disorders are treated by GPs exclusively and are not seen by specialists [15].

Within the specialist field of psychiatry, there is concern regarding over-diagnosing [16]. Some authors pose the question of whether the harm of psychiatric over-diagnosis may outweigh the benefits [17, 18] and whether physicians may struggle to distinguish between mental illness and typical behavior [19, 20].

In contrast in the past, studies addressed whether or not GPs under-diagnose psychiatric diseases [21–23].

The German healthcare system operates on a social insurance model, funded through dedicated contributions [24]. Depending on their income, patients have the freedom to choose between statutory and private health insurance companies, as well as their preferred physicians in primary (general) or secondary (specialist) health care sector. Consultations with GPs or psychiatrists are covered by the respective insurance. The doctors’ remuneration is determined primarily by the contact with the patient and is financed by the Association of Statutory Health Insurance Physicians [5]. The amount of money that might be earned is budgeted for each specialty. However, diagnoses nonetheless are an important justification for billing a contact. In Germany, psychiatric diagnoses are coded under the International Statistical Classification of Diseases and Related Health Problems (ICD-10) [25], commonly referred to as “F-diagnoses”.

One obvious way to explain differences in experiencing over- or under-diagnosing from the perspective of a different specialty is that diagnostic and testing procedures used in the specialized field of psychiatry are not directly transferable to the prevalences in primary care. Since the prevalence of psychiatric disorders is considerably lower in the general practice setting, the false positive rate for screening tests is significantly higher [26]. For example when using a test validated in a specialized psychiatric setting in the more low-prevalence range of GPs, only one third of those diagnosed by the instrument are actually ill [23].

There is a considerable amount of literature dealing with the topic of psychiatric diagnoses per se (see additional file 1 in **supplement**).

However, to meet the demands in mental health care, a profound knowledge about prevalences is necessary. Apart from specific research projects, routine care data to analyze the prevalences of diseases are still in their infancy in Germany [27–29].

The goal of this study was to identify reasons and motives for giving or rejecting psychiatric diagnoses in the different settings of GPs and psychiatrists. The rationale for this was that with a better understanding of the process of diagnosis, the understanding of routine care data as a source for the assessment of prevalence of psychiatric diseases as well as designing studies will improve.

## Methods

### Design

To identify theoretical concepts from the literature, publications related to the topic were screened (for details see additional file 1 in **supplement**) and complemented by searching cross references, internet browsers and e-books.

The following basic hypotheses were the basis of the semi structured guiding questions:

1. In addition to identifying a medical condition or illness, other factors play a qualitatively relevant role in the diagnosis of psychiatric disorders [30].
2. There are also specific reasons for not making such diagnoses, even though they would be indicated from a medical point of view [31].

The questions of the interview guide were therefore derived from informed concepts in the literature search as seen in the additional column in Table 1 and complemented by the authors own experiences (HTVE, medical student and doctoral thesis candidate; CN, MD and GP trainee; JS, GP and experienced in qualitative research).

**Table 1:** Semi structured interview questions

	Guiding questions	Theoretical background
1.	Why do you make diagnoses at all?	
2.	In which situations do you use diagnostic tools such as depression screening questionnaires?	[23]
3.	Are there mental disorders where you feel rather uncertain about the diagnosis? If so, why is that?	[32]
4.	In which cases do you not document mental disorders?	[31]
5.	To what extent does the potential stigma of a mental disorder influence you in documenting your diagnosis?	[8, 33]
6.	To what extent does the duration of the symptoms influence the diagnosis?	[34, 35]
7. *	To what extent does a patient’s continuity of care/experienced anamnesis influence the diagnosis?	[34]
8.	To what extent does the time pressure factor influence the diagnosis?	[36]
9.	How strongly do you feel the pressure for quick diagnosis from the patients?	[37]
10. *	How do you use the option of a referral for diagnosis?	[15, 37]
11.	In your experience, what are the differences between diagnostics in general medicine and psychiatry?	[14, 21]
12.	What other aspect of this topic that we haven’t touched upon today is important to you?	

Key: * These questions were only asked of GPs

The interview guideline covered aspects that might influence the process of assigning a psychiatric diagnosis. Open questions were formulated to allow detailed and rich responses by the physicians.

The interview guideline was revised by two experienced researchers in the field and finalized to include 12 questions (for details see Table 1).

We conducted semi-structured interviews focusing on the dynamics of physicians’ diagnostic processes in generating psychiatric diagnoses. For this study we let ourselves be guided by the COREQ-checklist (Consolidated Criteria for Reporting Qualitative Studies). The motives behind those dynamics were explored using the principles of the qualitative content analysis as described by Mayring [38].

### Participant selection and recruitment

Physicians from the fields of psychiatry and general medicine were informed about the option to participate in the study both verbally (e.g. at the Congress of the German College of General Practice and Family Physicians in the year 2021) and by mail via a convenient network of colleagues in three German states (Baden-Württemberg, Bavaria, Schleswig-Holstein). Apart from this convenient sampling approach, a snowball system was also used. The main aim was to reach an equal number of psychiatrists and GPs.

### Interview procedure

Telephone interviews were conducted with all participants who got in touch and who signed and returned the consent forms and met the inclusion criteria, such as medical qualification as GP or psychiatrist according to the German medical system and sufficient knowledge of German for the purpose of the interview. The telephone interviews took place from June to December 2021. They were performed by the same researcher (HTVE), who was trained in interviewing by the Institute of Family Medicine in Lübeck. Interviews were recorded with a voice recorder (“Voice Tracer DVT6110”), pseudonymized and transcribed using the transcription software f4 (version 4.2.). Three of the participants knew HTVE. In order to standardize the transcripts, they were created according to the transcription rules of the Institute of Family Medicine at the University of Lübeck. The interviews conducted were scheduled to last 30–45 min.

### Data analysis

Subsequently, the transcripts were analyzed based on the principles of content analysis according to Mayring [38]. The participants quotes were cited to illustrate the results and each quote was identified by a pseudonymized number. The concepts of the interview guide were predetermined and therefore the first step of the analysis was deductive.

The essential content passages were identified and assigned to a main category as a coding unit. The coding unit was defined as a new subcategory or assigned to an existing subcategory. The main categories and subcategories were modified during the process if necessary to improve comprehensibility. It was also possible to develop new main categories and subcategories inductively. At the end of the process, a coding system with new main and sub-categories and corresponding anchor quotations was created. This process was carried out by two scientists independently of each other (HTVE & CN).

Once the development of the category system was complete, all the material marked as relevant in terms of content was reviewed again to ensure that no important content was overlooked in the structured summarization processes. At the end of the evaluation process, the independently created category systems were compared and discussed in a group process with an additional scientist (JS). In several meetings, both systems were discussed in detail, debated and merged in order to develop a final consensus version of the category system. In the course of these sessions, the different categories were assigned to four different thematic areas, which were derived from the main categories and guiding questions.

### Ethical considerations

The Ethics Committee of the University of Lübeck approved the ethics application in April 2021 (Az. 21–120).

## Results

12 GPs and 14 psychiatrists took part in the study. Five physicians initially interested in the study did not participate, giving no reason. 54% of the participants were female with the average age of the participants being 54 years. On average, participants had 24 years of work experience. Further details of the demographics of our participants are presented in Table 2.

**Table 2:** Demographic data of study participants compared to physicians in the whole of Germany [39, 40]

Sociodemographic variable	Mean value of all participants	Mean value of GPs	Mean value of psychiatrists	Mean value of GPs in Germany	Mean value of psychiatrists in Germany
Mean age (in years)	54.3	51.0	57.1	55.3	54.5
Work experience (in years)	24.3	21.3	27.0		
Sociodemographic variable	Number (n), Percent (%) all	n (%) general practitioners	n (%) psychiatrists		
Sex (f : m) (f %)	(14:12) (54%)	(6:6) (50%)	(8:6) (57%)	58%	63%
Urban practice	24 (92%)	11 (92%)	13 (93%)		
Rural practice	1 (4%)	1 (8%)	0		
Practice location not specified	1 (4%)		1 (7%)		

The interviews lasted 30 min on average. GP interviews lasted 32 min on average (between 19 and 53 min) and psychiatrist interviews lasted 27 min (between 20 and 36 min).

The content analysis of all interviews revealed four major thematic domains: (i) Motives and modifying factors for giving or rejecting a psychiatric diagnosis, (ii) Methodological aspects of finding a diagnostic conclusion, (iii) Subjectively perceived diagnostic and therapeutic expectations in the medical system (physician’s perspective) and respective interprofessional cooperation issues, and (iv) Expectations of patients with psychiatric symptoms in the general medicine and psychiatric setting.

**Fig. 1:**
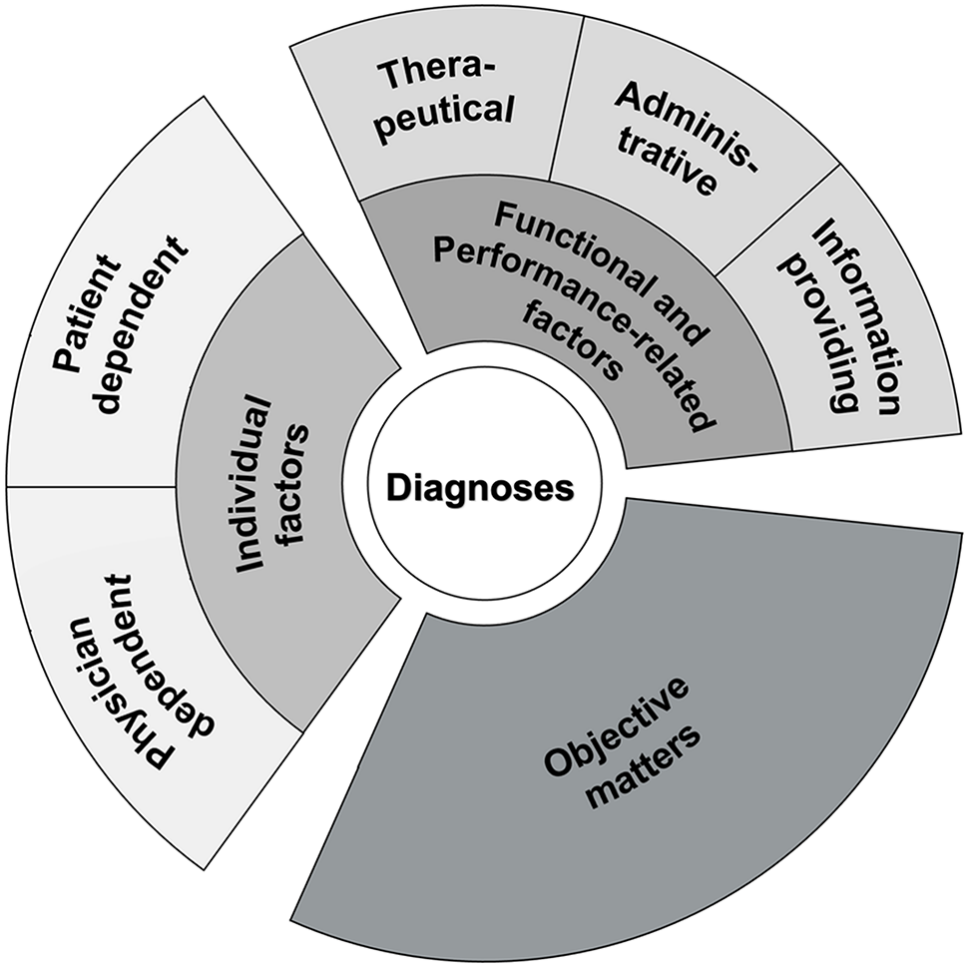
Motives and modifying factors that play a role for giving or rejecting psychiatric diagnoses. Model consisting of main and sub-categories of qualitative content analysis

In this paper, we concentrate on the results of the first domain to focus on our main research question. In this domain, we identified three main categories in the process of giving or rejecting a diagnosis with five subcategories illustrated in Fig. 1 and detailed with several codes in Table 3.

**Table 3:** Analytical category system of the content analysis including main categories, subcategories and codes

Main category	Subcategory	Code
*Motives for diagnosing*		
Objective matters		Designation of a fact
		Categorisation for scientific knowledge and international communication
		Differentiation of clinical pictures
		Classification scheme
Functional and performance-related factors	Functional therapeutic factors	Basis of treatment
		Requirement for medication
		Requirement for therapy
		Classification of symptoms/ Patient education
		Prognosis/ Realistic expectations of therapy
		Relief of the patients
	Functional administrative factors	Billing aspects
		Financial Incentivization of diagnoses
		Requirements from health insurance companies/ other insurance companies to make diagnoses
		Requirement for referral to specialized centre
		Possibility of documentation
		Access to adequate care
	Information providing factors	Communication with colleagues
		Communication with patients
		Relief for relatives
Individual factors	Individual physician-dependent factors	Personality of the physician e.g. intrinsic demand to always make a diagnosis
		Self-image of the physician e.g. tolerance towards psychiatric diagnoses
		Experience as a physician
		Expertise of the physician
		Physician’s generation
		Education of the physician
		Relief for therapists (aggressive response to frustration experience)
	Individual patient-dependent factors	Age of the patient
		Previous diagnoses of the patient
		Personality of the patient e.g. physician hopping
		Intellectual status and explanatory models of the patient
*Motives for not diagnosing*		
Objective matters		Inaccuracy of psychiatric diagnoses
		Problems in differentiating between pathological and normal symptoms
Functional and performance-related factors	Functional therapeutic factors	Assumption that knowledge of psychiatric diagnosis does not imply additional benefit for co-therapists
		Assumption that knowledge of psychiatric diagnosis does not imply additional benefit for the patient
	Functional administrative factors	Juridical consequences in case of e.g. suicidality of the patient
		Differences in insurance status private or non-private
		Psychiatric diagnosis permanently in the patient’s file
		Detrimental insurance status with psychiatric diagnosis
		Patient’s desire to become a civil servant
		Patient’s wish to take out a loan
		Rehab request of the patient
Individual factors	Individual physician-dependent factors	Preservation of confidentiality
		Fear of stigmatisation among patients
		Fear of negatively biasing other practitioners
		Fear of negatively affecting the physician-patient relationship
		Lack of knowledge of the clinical picture
	Individual patient-dependent factors	Patient’s request not to have a psychiatric diagnosis
		Recognised clinical picture is not the reason for the patient’s consultation
Alternatives to not diagnosing		Graded scoring of psychiatric diagnoses
		Coding of other clinical pictures
		Referral to others for diagnostic purposes
		Reframing of diagnostic terms

### Motives for diagnosing psychiatric disorders

Three different main categories could be identified that positively motivated physicians to attribute a psychiatric diagnosis:

### Diagnosis as an objective matter

In our inductive analysis process, we defined diagnosis as an objective matter related to the determination of a fact. When asked why diagnoses were allocated, some of the responses addressed its objective nature.

In general, diagnoses serve to categorize patients, i.e. disease patterns, in order to distinguish them from one other. Other physicians mentioned that generating a diagnosis is indispensable for international research and communication.

*“The aim is to form groups that are as uniform as possible so that they can be researched in the broadest sense.” P11 (P = Psychiatrist)*.

### Functional and performance-related factors

Functional and performance-related factors refer to different functions resulting from the diagnosis. In this context, it means that the content of the function has an intended mode of action or purpose.

### Functional therapeutic factors

Most importantly, the diagnosis was seen as the basis for treatment by some physicians. This means that the diagnosis implicated specific action following guidelines and recommendations for the physicians like medications, illness prescriptions, psychotherapy and much more. Especially the sub-category of diagnosis as a condition for medication was mentioned frequently. In some statements physicians pointed out that there were situations where they were not convinced of the correctness of the diagnosis but still needed it in order to prescribe a certain drug.

*“Or he has anxiety attacks that I do not yet consider sufficient to diagnose an anxiety disorder, but feel that I want to give him something for the exam so that he can simply pass it and get over this hurdle, so I make the appropriate diagnosis so that I can prescribe the medication. So that’s a functional diagnosis, if you like, and not a factual one.” P4*.

### Functional administrative factors

Administrative tasks involve overseeing and organizing personal affairs or those of someone else, typically within a structured setting such as government agencies or organizations, in this case within the health care system as well as with insurance companies.

A related point was the requirement of a diagnosis for billing purposes from health insurance companies or the Ambulatory Health association, which practically forces physicians to make a diagnosis. Many physicians emphasized that they often felt they had to diagnose diseases they were not convinced of because of this billing pressure.

*“Because you are really forced by the system to at least commit to one (…) so you can’t write: he came to see me but I don’t know what he has. That means you can’t bill for that.” P14*.

*“You can write a suspected diagnosis first. But after a quarter, i.e. after three months, the KV demands that you check it and make a confirmed diagnosis or drop it.” P13*.

(KV ◊ Association of Statutory Health Insurance Physicians)

In the German healthcare system some diagnoses generate more money for the treating physician than other diagnoses, which can lead to incentivization of specific diagnoses.

*“And then there was a figure that implied money per patient per quarter if the patient had certain rather more serious diagnoses. Of course, this has, how shall I say, given a slight distortion to the more severe diagnoses.” P1*.

Some GPs emphasized the great influence of administrative needs of insurance companies on allocation of a diagnosis, one example being disability insurance.

*“So if someone is unable to work because of a mental illness and this drags on for a certain period of time, then this also forces a diagnosis.” GP2*.

However, health insurers also required psychiatric diagnoses for certain services, which led physicians to make them.

*“I can’t code it under a flu-like infection or normal exhaustion R53 for example, it wouldn’t get waved through, so it really has to be an adjustment disorder, it has to be, yes exactly, it has to be an F-diagnosis, so that the health insurance company says: “All right, it’s justified, we’ll pay for the psychotherapy”.” GP12*.

### Information-providing factors

The term “information-providing factors” refers to the social or informative benefit for the recipients of the diagnosis, which is created by passing on the diagnosis and the information it contains. This means that the diagnoses include different treatment or therapy options, which can be helpful when communicating with other colleagues, but also with patients, and can help the patient understand more quickly.

*“So if I say: “Someone has schizophrenia”, this is different from me saying to a colleague: “He has severe depression with psychotic symptoms”, for example. I think both have psychotic symptoms, but they have a different status and therefore a different value in treatment.” TNP10*.

Another aspect mentioned by the participants was that a psychiatric diagnosis can be a relief not only for the patient but also people around them, such as friends and partners, since the diagnosis may help them understand the patient’s behavior better. As an example, one physician spoke about the relieving effect of a diagnosis for the patient’s relatives, who as a consequence no longer blamed themselves.

### Individual factors

Individual factors do not primarily relate to objective facts or specific functions of a diagnosis, but rather to individual framework conditions across situations.

### Individual physician-dependent factors

In the interviews, it became apparent that there were also diagnostic styles that differed not only between the two specialist domains, but also within them. Some physicians justified this different way of thinking about and making diagnoses with individual differences of the physicians themselves. As an example, the individual personality of the physician was mentioned. Psychiatrists in particular seemed to have an intrinsic claim to make a diagnosis.

*“When I interview someone and want to find out what they have, then for me, somehow the requirement is that I want to have a diagnosis” P5*.

Other structural differences, such as the self-image of the physician to be tolerant towards psychiatric diagnoses, also had an impact on making a diagnosis. Other individual factors were experience and expertise, as well as the generation of the physician.

*“For example, with young general practitioners (…) I have the feeling that it is different, that they are already more informed about (…) that psychiatric diagnoses are just more of an option.” P2*.

The school of training also had an effect. In some universities, for example, some diagnoses were categorically excluded, which led the physicians to continue this practice in their later work.

*“Well, I grew up as a purely behavioral psychiatrist. Also because I studied in < city>, where psychiatrists and psychosomatics are, at least on the face of it, mortal enemies. And as a consequence, I diagnose very few of these so-called somatoform disorders.” P11*.

One psychiatrist described a situation where other psychiatrists had given a diagnosis of a personality disorder to a difficult patient because they themselves were frustrated with the treatment, and diagnosing a personality disorder offered an excuse for the treatment failure.

*“I have also often experienced that specifically borderline disorders, or also narcissistic personality, personality disorders – were given, more as a reaction to the annoyance of dealing with the patient for weeks, that you didn’t get on properly: “Ah, he must have a personality disorder.”” P11*.

### Motives for not diagnosing

It also became apparent that there were several motives for not attributing diagnoses.

### Objective matters

Many participants criticised the inaccuracy of psychiatric diagnoses as they often represent a mixture of norm variants and disease-like conditions. The different classification systems of the ICD and the Diagnostic and Statistical Manual of Mental Disorders (DSM) and their different categorisations were often cited as evidence of the model-like nature of the diagnostic categories. Syndrome diagnoses, which are common in the psychiatric field, were often taken up as a construct.

*“Diagnoses are always, especially in psychiatry (…) almost always syndrome diagnoses, which means that there is often a certain vagueness in it and it is always a construct.” P8*.

Many physicians mentioned the problem that there are no intermediates between normal and pathological ratings in the coding systems. This led to physicians assigning diagnoses that they themselves doubted were real in order to obtain certain services for patients. Some physicians wanted alternative diagnoses that would better reflect these intermediate states.

*“If I think that the patient has a condition that, unfortunately, if it were coded, could be a mental disorder, I mean now like a grief reaction or, you can be in a bad mood, that this is then immediately an F-diagnosis, there is nothing that can be coded so reasonably.” GP12*.

Some stated that many clinical pictures to be diagnosed according to the guidelines were often explainable and normal in the individual context. However, the health system would often turn this into a disease by forcing a diagnosis, even though physicians would normally regard this as a normal variant of health.

### Functional and performance-related factors

#### Functional administrative factors

Some psychiatrists reported that insurance status also influenced whether or not the diagnosis was made. E.g. as patients being privately insured may receive their coded diagnosis with their invoice directly after contacting the physician for billing purposes together with the report on diagnostic findings. Whereas this is not the case for patients with a statutory health insurance.

Many physicians reported that they were cautious when making diagnoses, as the diagnosis could no longer be removed from the insurance companies’ patient records. Access to certain insurances, such as occupational disability or life insurance, could be more difficult or no longer possible. In addition, certain diagnoses can make it more difficult or even impossible to take out a loan or to become a civil servant.

*“Then, of course, a confirmed diagnosis from my side would have consequences, not only in terms of stigmatization, but also, for example, for insurance companies or something like that later on. Once something like that is in there, it’s hard to get it out again.” GP2*.

### Individual factors

#### Individual physician-dependent factors

The issue of stigmatization played a major role in the interviews. Physicians from both specialist areas reported that the fear of stigmatization influenced them when making a diagnosis. First, some physicians mentioned that especially the patients were afraid of experiencing stigmatization. At the same time, however, there was also mentioning of potential prejudice by other physicians if they were to read the diagnosis in the patient’s file.

*“Let’s say a physiotherapist gets a prescription for physiotherapy and it says: F45.1 chronic pain disorder with psychological stress, then this might prejudice them against the patient.” GP12*.

### Alternatives to not diagnosing psychiatric disorders

Because of the above-mentioned stigmatization on many levels, alternative ways of dealing with diagnoses were reported in the interviews. Often, for example, a more harmless diagnosis from the psychiatric classification system was chosen instead of the more valid one:

*“Then I do try to merely classify it as an adjustment disorder, which is of course also a psychiatric diagnosis, but certainly the least disabling one for someone when it shows up in the health insurance documentation.” P8*.

Sometimes, however, the physicians chose a completely different diagnosis in order to guarantee the functional goal, e.g. sick leave.

*“If I have a student who comes to me with love sickness, then I tell him quite clearly: “I’ll give you (…) a sick note. But I’ll put down stomach pain or flu.” GP12*.

## Discussion

This study supports the assumption that the diagnostic process is a complex phenomenon that goes far beyond the identification of medical facts. It suggests that routine care data, derived from the primary and specialist care system, do have important limitations. This insight needs to be considered when interpreting secondary data.

In our study, three different categories of motives for the diagnosis or non-diagnosis of psychiatric disorders were identified (objective factors, functional and performance-related factors and individual factors).

It is already known that diagnoses may also depend on other factors such as collegial norms, economic incentives, prodigious traditions or expectations of patients [14]. Psychodynamic and phenomenological elements can also play a role in the process between physician and patient when making diagnoses [15]. Therefore, the diagnostic process is much more complex and includes many facets, amongst others the need to understand patients [3, 41–44].

This is an important limitation regarding the understanding of prevalence provided by routine care data in scientific literature. When reading international classification systems such as the ICD-system or the DSM-system [45, 46], different S3-guidelines [47] or standard medical textbooks [35], reference is basically made to the presence or absence of medical findings when diagnostic entities are operationalized. However, the results of this study suggests that this is not always the case. This illustrates that it might be problematic to regard diagnostic data deriving from the primary and spezialized medical care system as representing true medical fact. The different views on over-diagnosis in specialized psychiatry and under-diagnosis in general medicine mentioned at the beginning [9, 16, 17, 19, 22, 23] could thus be put in a different light. Our results suggest that physicians might be exposed to different influences such as individual or administrative aspects that lead them to make or not make certain diagnoses. Future studies should also address whether the experienced prevalence leads to an additional bias regarding the impression of under- or over-reporting of diagnoses.

A major topic in the interviews was functional and performance-related factors and in particular administrative functions of psychiatric diagnoses. Participants mentioned incentivization and billing implications of a diagnosis, but also referred to it as being a precondition before many health insurance companies will offer certain patient support services. This observation is in line with similar propositions in the literature, where implications of diagnoses for pensions, disability certificates, assessments of degrees of disability, death certificates etc. have been implicated [41]. It also became clear that individual factors such as individual physician-dependent factors also have an impact on the diagnostic process. This is in line with an Australian study with GPs which also found that diagnoses are highly dependent on individual characteristics of the physicians, such as age, sex and practice organization [48]. Other studies point out that patient-dependent factors like sex and race may also influence the diagnosis [11, 12].

Among the motives found against making a diagnosis, the individual physician-dependent one was particularly prominent. Here, fear of stigmatization at various levels was mentioned many times, which led physicians to either not allocate diagnoses at all or to deviate and trivialize them. The literature confirms the feared stigmatization in various areas of social reality [7, 49, 50] such as the workplace [51, 52] as well as experiences of discrimination in different areas of life and interpersonal relationships [53]. Another study found that physicians too exhibit stigmatizing behavior towards people with psychiatric disorders [33]. Yet another factor speaking against making psychiatric diagnoses were administrative factors. In this context the negative economic consequences of psychiatric diagnoses, which result from structural disadvantages in the regulations of insurance policies such as private life, health or occupational disability insurance have been pointed out [54]. In addition, it might well prove impossible to enter certain areas of civil service or specific professions like jobs in the police with such conditions.

It must also be emphasized that some participants reported that higher billing figures for certain diagnoses led them to code more severe diagnoses. However, not all countries rely on a multitude of health insurances, for instance Denmark, Sweden, and Norway. Given these obvious far-reaching detrimental consequences of psychiatric diagnoses, it is remarkable that there is still very little literature on this topic complex. More research is needed to better understand the impact of administrative factors like the incentives of the healthcare system and the influence of insurance conditions on making diagnoses.

If there are differences between the two medical specialties in the way of making a diagnosis, as suggested by research by Davidson and Fosgerau [21], this should be addressed in future studies.

### Strengths and limitations

According to our literature search and to the best of our knowledge, to date there are no comparable studies that have explicitly and empirically dealt with the motives and modifying factors for giving or rejecting psychiatric diagnoses in the settings of psychiatric and GP out-patient care. In terms of socio-demographics, the gender and age distribution of the participants in this study is comparable regarding physicians and their specializations in Germany. Selection bias cannot be ruled out as a convenient sampling method was used. Therefore, participants who had already dealt intensively with the topic of psychiatric diagnoses might have rather participated. The interviewer was familiar with three participants which may have potentially influenced the interview dynamic.

Further quantitative studies could build on our suggested categorical model and extend it to analyze the role of socio-demographic factors or items like further specializations or places of medical training in detail. Due to the methodological limitations, no conclusions can be drawn for Germany or other countries since people in different geographical, social or cultural settings may face different structural and social realities and thus think differently about diagnosing. We can’t rule out that using a different qualitative approach other than telephone interviews e.g. face to face would have led to additional information.

Finally, all researchers are working in the field of family medicine, which might be a source of bias within the analysis due to their generalist perspective.

## Conclusion

This study adds further weight to the notion that the diagnostic process is a complex phenomenon that goes far beyond the identification of medical facts or categorical diseases, which is especially clinically important for post-graduate trainees.

We were able to identify three different categories of motives to diagnose or not to diagnose psychiatric disorders (objective matters, functional as well as performance-related factors and individual factors). As a consequence, this may indicate that routine care data should be treated with a degree of caution if used to draw conclusions regarding health care demands or study design.

Also, given that psychiatric diagnoses affect the self-image, identity and self-worth of people much more than most other medical diagnoses, this insight is of utmost importance in general medicine and psychiatry. Stakeholders in the medical system should contemplate this and realize that the diagnostic process also depends on which sector of the medical system they work in.

### Electronic supplementary material

Below is the link to the electronic supplementary material.

Supplementary Material 1

## Data Availability

The dataset used and/or analysed during the current study are available from the corresponding author on reasonable request.

## Abbreviations

DSM: Diagnostic and Statistical Manual of Mental Disordersl
GP: General practitioner
ICD: International Statistical Classification of Diseases and Related Health Problems
KV: Kassenärztliche Vereinigung = Association of Statutory Health Insurance Physicians)
P: Psychiatrist
