# Supplementary material for: Motives and modifying factors for giving or rejecting psychiatric diagnoses in general medicine and psychiatry – a qualitative interview study
Source: BMC Psychiatry. 2024 Jun 20;24:461. doi: 10.1186/s12888-024-05900-2 (PMC11188242; doi:10.1186/s12888-024-05900-2)
Supplement: Supplementary file 1 — Supplementary Material 1 [file 12888_2024_5900_MOESM1_ESM.docx]

**Additional file 1** Illustration of systematic research strategy via PubMed

| Search terms | Search location | Citations identified | Relevant citations following analysis at abstract level | Reference | Content of paper |
| --- | --- | --- | --- | --- | --- |
| Psychiatric diagnosis AND general medicine | All fields | 58089 | Not analysed |  | |
| (psychiatric diagnosis[Title]) AND (general medicine[Title]) | Title | 0 |  | | |
| Psychiatric diagnosis (title) AND general medicine (all fields) | title and all fields respectively | 43 | 3* | (2)  “Culture and psychiatric diagnosis. Impact on DSM-IV and ICD-10” | Theoretical reflection on the impact of culture on DSM-IV and ICD-10 |
|  |  |  |  | (50) Psychiatric diagnosis in primary care patients with increased depressive symptoms | Prevalence study of psychiatric diagnoses in a general medical setting |
|  |  |  |  | (3)  Patterns of psychiatric diagnosis in general practice: the Second National Morbidity Survey | Large survey pointing out that diagnostic habits of physician are relevant in diagnostic process |
| (((psychiatric diagnosis) AND (general medicine)) AND (psychiatry)) AND (motives for diagnosing) | All fields | 315 | 2* | (37) „Investigating the impact of primary care payments on underdiagnosis in dementia: A difference-in-differences analysis“ | Large survey that found out that financial incentives can lead to improved performance in primary care |
|  |  |  |  | (51) „Comparison of DSM-IV and DSM-5 criteria for alcohol use disorders in VA primary care patients with frequent heavy drinking enrolled in a trial“ | Study pointing out that when changing the classification system from DSM-4 to DSM-5 significantly more patients with alcohol use are identified and diagnosed than with the previous system |
| ((general medicine[Title]) AND (diagnosis[Title])) AND (psychiatry) | title and all fields respectively | 1 | 0 |  | |
| Psychiatric diagnosis | All fields | 1482499 | Not done |  |  |
| Psychiatric diagnosis | title | 840 | 11* | (36) Psychiatric diagnosis: personalised by definition | Review indicating that the diagnosis is aimed not only at etiological but also other factors such as the course and treatment of the disorder |
|  |  |  |  | (52) Prescribing of psychotropic medications to patients without a psychiatric diagnosis  (53) Proportion of antidepressants prescribed without a psychiatric diagnosis is growing | Surveys, claiming that lots of recipients of psychotropic or antidepressant medication lacked a clear indication (diagnosis) for such use |
|  |  |  |  | (10)  The effects of sociodemographic factors on psychiatric diagnosis | Clinical study, that found out that demographic factors, including ethnicity, have effects on diagnoses in psychiatric inpatients. |
|  |  |  |  | (54) Psychiatric diagnosis. A cultural perspective | Report outlining further factors that capture the disease diagnosis in a socio-medical frame of reference. |
|  |  |  |  | (35)  Advances in Psychiatric Diagnosis: Past, Present, and Future | Study highlighting that diagnoses continue to have essential purposes, including selecting the most effective treatment, communicating with colleagues about diseases, educating about psychiatric disorders, and supporting ongoing research. |
|  |  |  |  | (55)  Culture, cultural factors and psychiatric diagnosis: review and projections | Paper focusing on the the role and relevance of culture in the diagnostic encounter |
|  |  |  |  | (56) Psychiatric diagnosis and nosological theory: studies on the individual diagnostic schema of the physician | Study, highlighting that the psychiatric diagnostic scheme determines the clinical diagnosis in everyday practice, whereas the internalized nosological theory is of importance mainly in research |
|  |  |  |  | (11)  Effects of race on psychiatric diagnosis of hospitalized adolescents: a retrospective chart review | Review pointing out that patient race and sex may influence clinical psychiatric diagnoses of hospitalized adolescents |
|  |  |  |  | (12)  Racial and sexual bias in psychiatric diagnosis: psychiatrists and other mental health professionals compared by race, sex, and discipline | Study, pointing out that therapists appeared more likely to make judgments biased against patients who were of the same race and sex as themselves. |
|  |  |  |  | (57)  The dependence of psychiatric diagnosis on the function of the diagnostician in relation to his patient | Study saying that physicians always tend to observe and assess the sides of the disease that affect their particular area of responsibility first. |
| Psychiatric diagnosis AND motive | All fields | 45195 | Not done |  | |
| Psychiatric diagnosis AND motive | title | 0 | 0 |  | |
| Motives AND diagnosis | title | 4 | 0 |  | |

**Legend: *** papers were only included when presenting an abstract and written in English
